# Supplementary figures and images for: Glycan cross-feeding supports mutualism between Fusobacterium and the vaginal microbiota
Source: PLoS Biol. 2020 Aug 25;18(8):e3000788. doi: 10.1371/journal.pbio.3000788 (PMC7447053; doi:10.1371/journal.pbio.3000788)

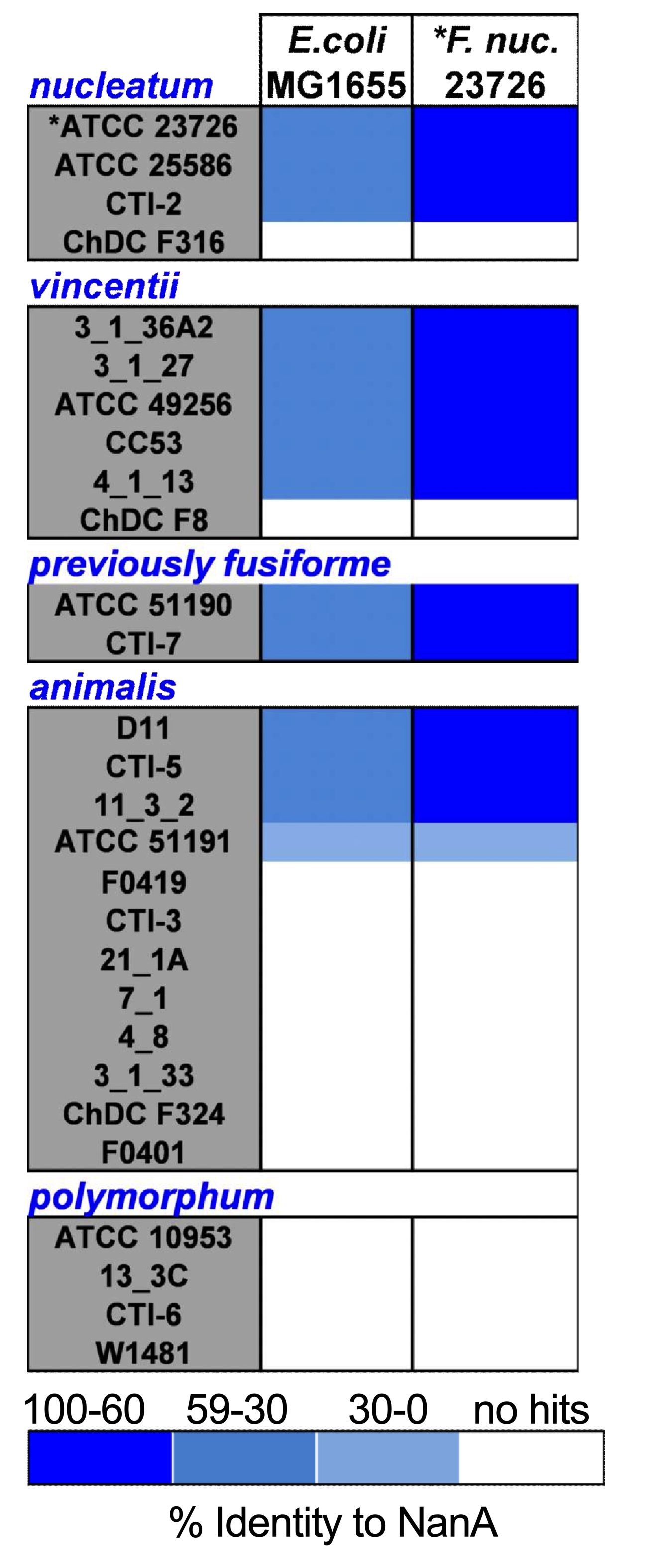

Supplement: S1 Fig — (A) Heat map showing percent identity of NanA homologs in sequenced F. nucleatum strains to the amino acid sequence of sialic acid lyase of E. coli MG1655. Homologs showed high similarity to amino acid sequence of lyase from F. nucleatum ATCC23726. ATCC, American Type Culture Collection; NanA, N-acetylneuraminate lyase. (TIFF) [file pbio.3000788.s001.tiff]

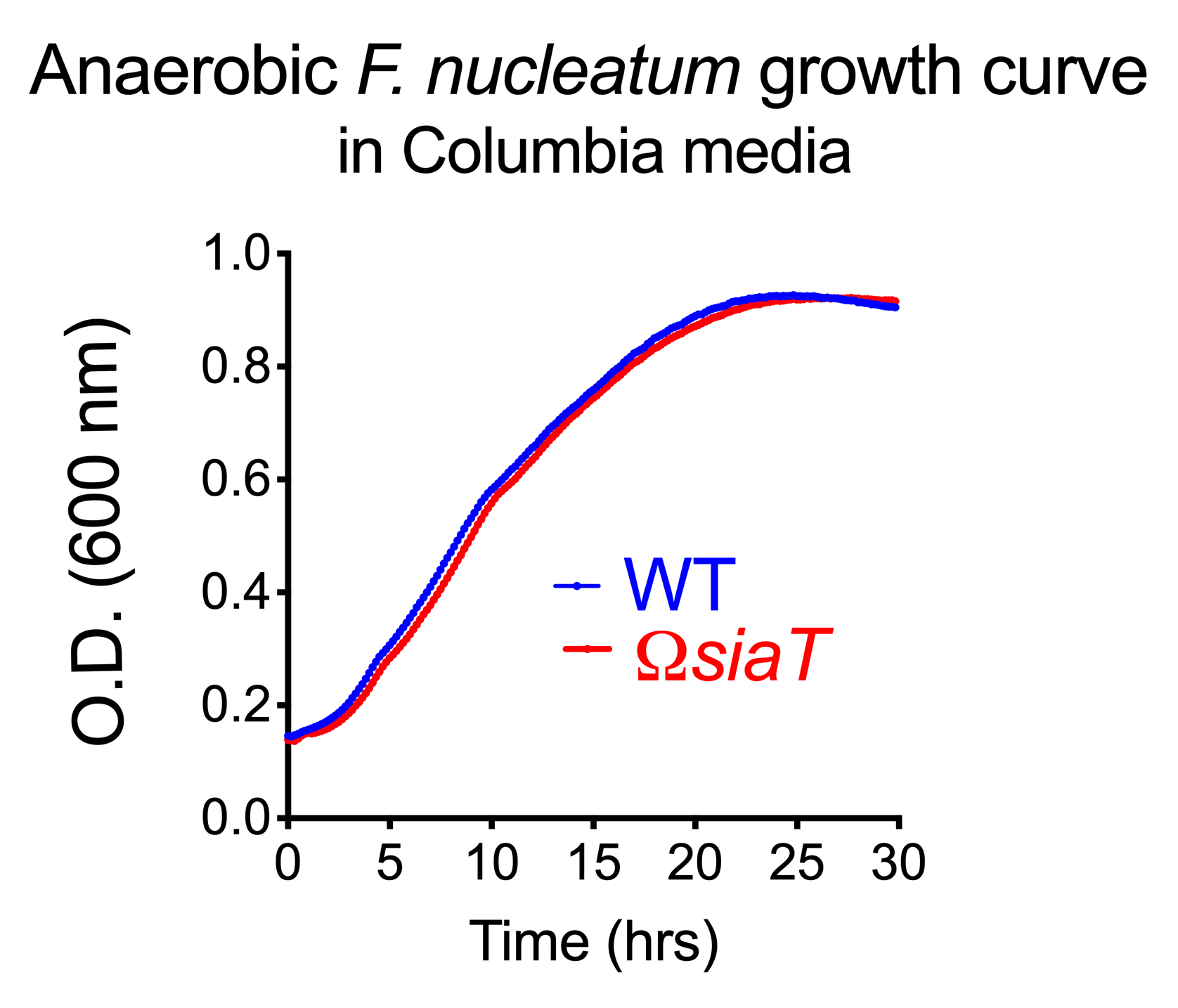

Supplement: S2 Fig — (A) F. nucleatum WT and ΩsiaT show similar growth when cultured anaerobically under these culture conditions. Data shown are representative of 3 independent experiments. siaT, predicted sialic acid transporter; WT, wild type. (TIFF) [file pbio.3000788.s002.tiff]

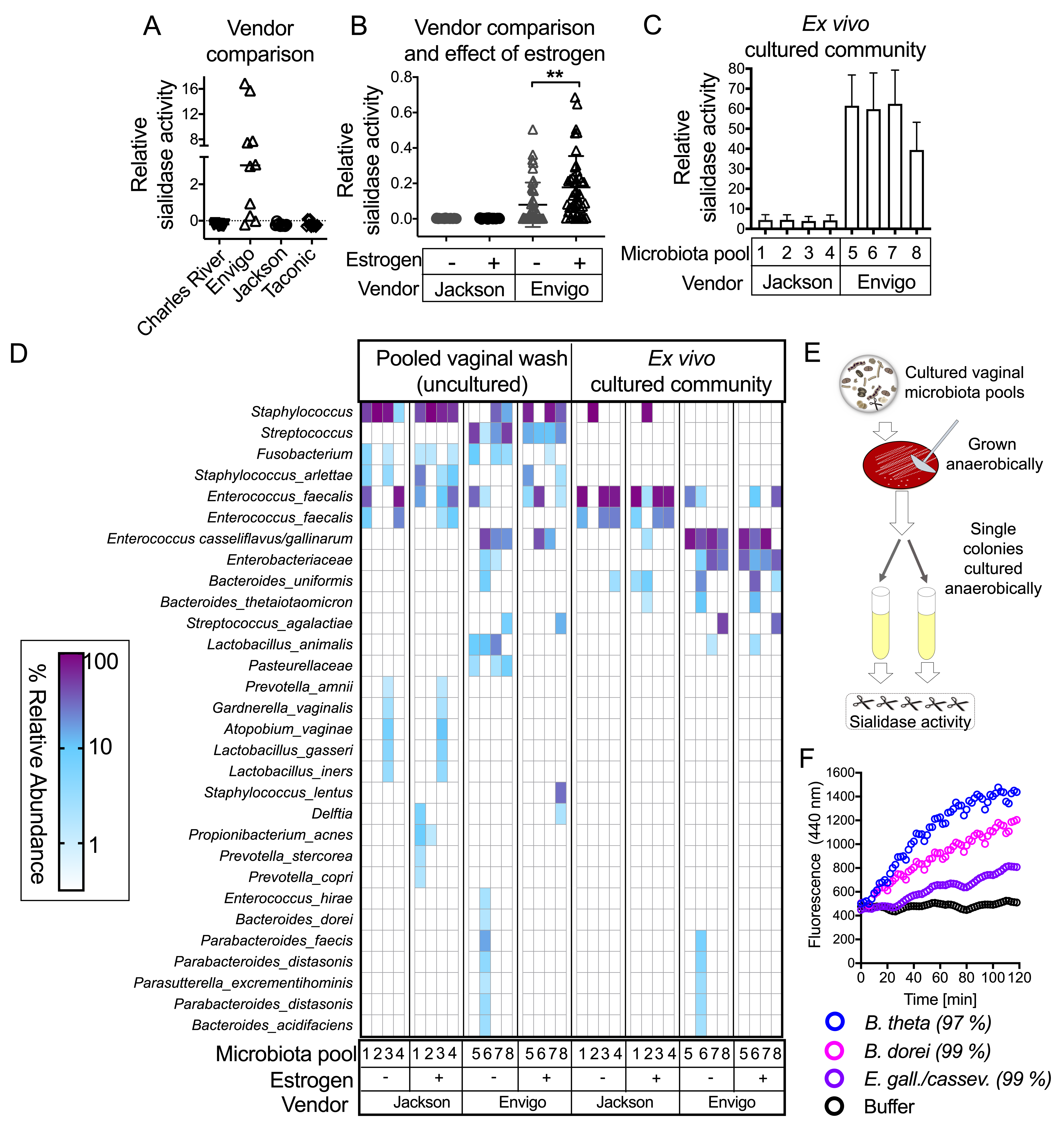

Supplement: S3 Fig — (A) Sialidase activity in vaginal washes of mice (not estrogenized) from different vendors measured using the fluorogenic 4MU-Neu5Ac substrate. N = 10 mice/vendor. (B) Vaginal sialidase activity is elevated at 72 h postestrogenization in mice from Envigo. No sialidase activity was detected in vaginal washes of mice from Jackson. N = 40 mice/vendor. Wilcoxon paired-sign rank test was used for pairwise comparison. **P < 0.01. (C) Sialidase activity in microbiota pools from Envigo and Jackson mice, collected after estrogenization. Each “microbiota pool” consists of a cultured vaginal community from pooled vaginal washes of 4–5 cohoused mice. For A, B, and C, data shown are combined from 2 independent biological replicates. (D) Heat map shows relative abundance of bacterial taxa identified by 16S rRNA V1–V2 sequencing in vaginal specimens of Envigo and Jackson mice collected before and after estrogenization. Microbiome analysis was done on uncultured and cultured vaginal washes pooled from 5 mice housed in the same cage. Each column represents 1 pool = 1 cage = 5 mice. Total = 4 microbiota pools per vendor per condition. OTUs were clustered using UPARSE-OTU algorithm, and taxonomic predictions were assigned using the RDP 16S rRNA database. (E) For identification of sialidase-positive bacteria in Envigo mice, frozen microbiota pools were streaked on supplemented Columbia blood plates anaerobically incubating for 24 h at 37°C. Single colonies were gently picked from these plates and cultured overnight. (F) Sialidase-positive cultures were identified using 4MU-Sia assay. Strains were identified by amplification and sequencing of 16S gene. Percent identity to the recovered blast hits is given in parentheses. The underlying numerical data for this figure can be found in S1 Data and S2 Data. Neu5Ac, N-acetylneuraminic acid; OTU, Operational Taxonomic Unit; RDP, Ribosomal Database Project; 4MU, 4-methylumbelliferone. (TIFF) [file pbio.3000788.s003.tiff]

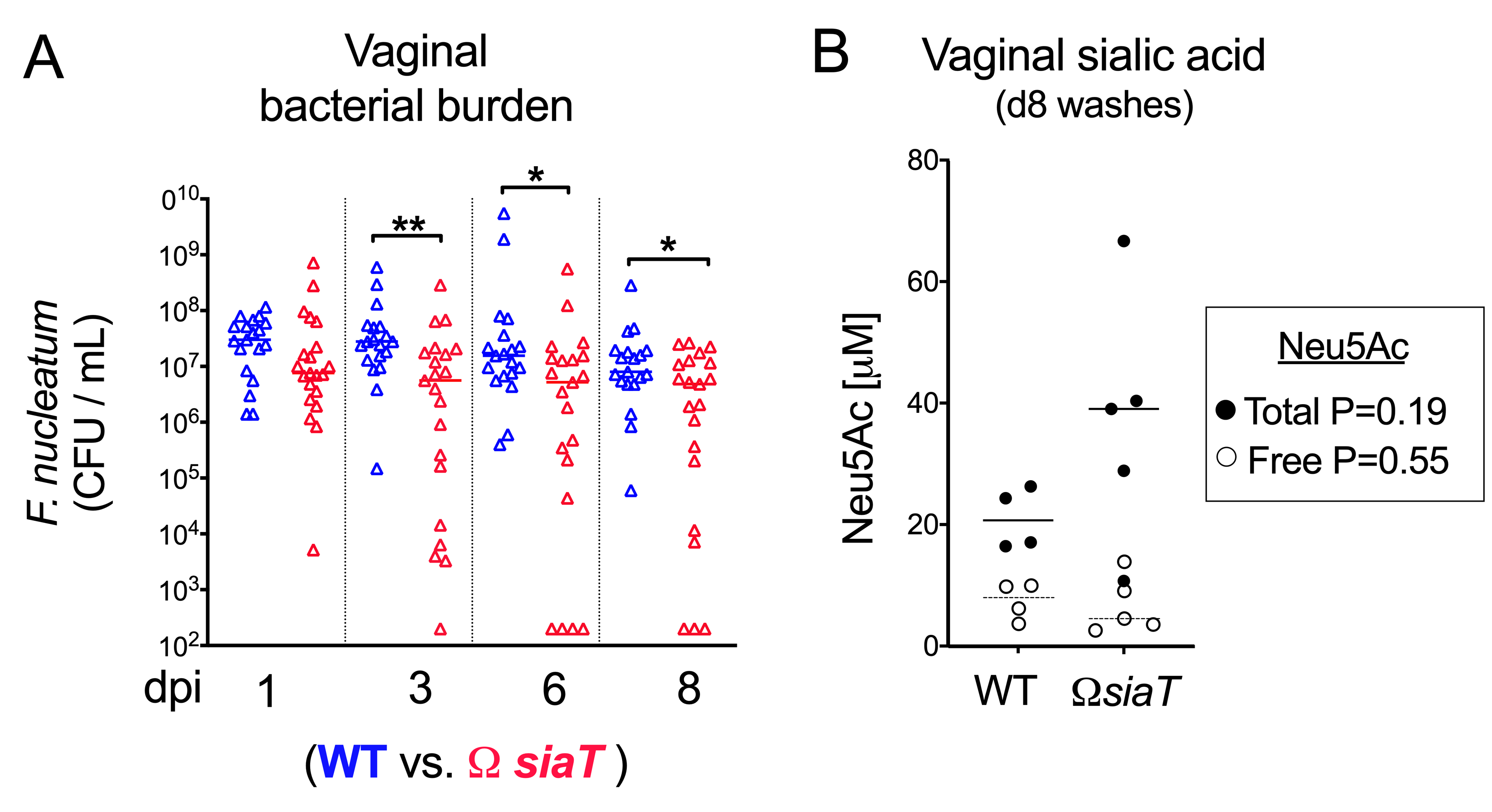

Supplement: S4 Fig — (A) F. nucleatum titers in vaginal wash collected at the indicated time points postinoculation. Data are combined from 2 independent experiments. Each experiment had 10 mice per group. *P < 0.05, **P < 0.01, Mann–Whitney. (B) Free and total sialic acid (Neu5Ac) concentrations in vaginal wash at 8 dpi. Total N = 18. The underlying numerical data for this figure can be found in S1 Data. dpi, days postinoculation; N-acetylneuraminic acid; siaT, predicted sialic acid transporter; WT, wild type. (TIFF) [file pbio.3000788.s004.tiff]

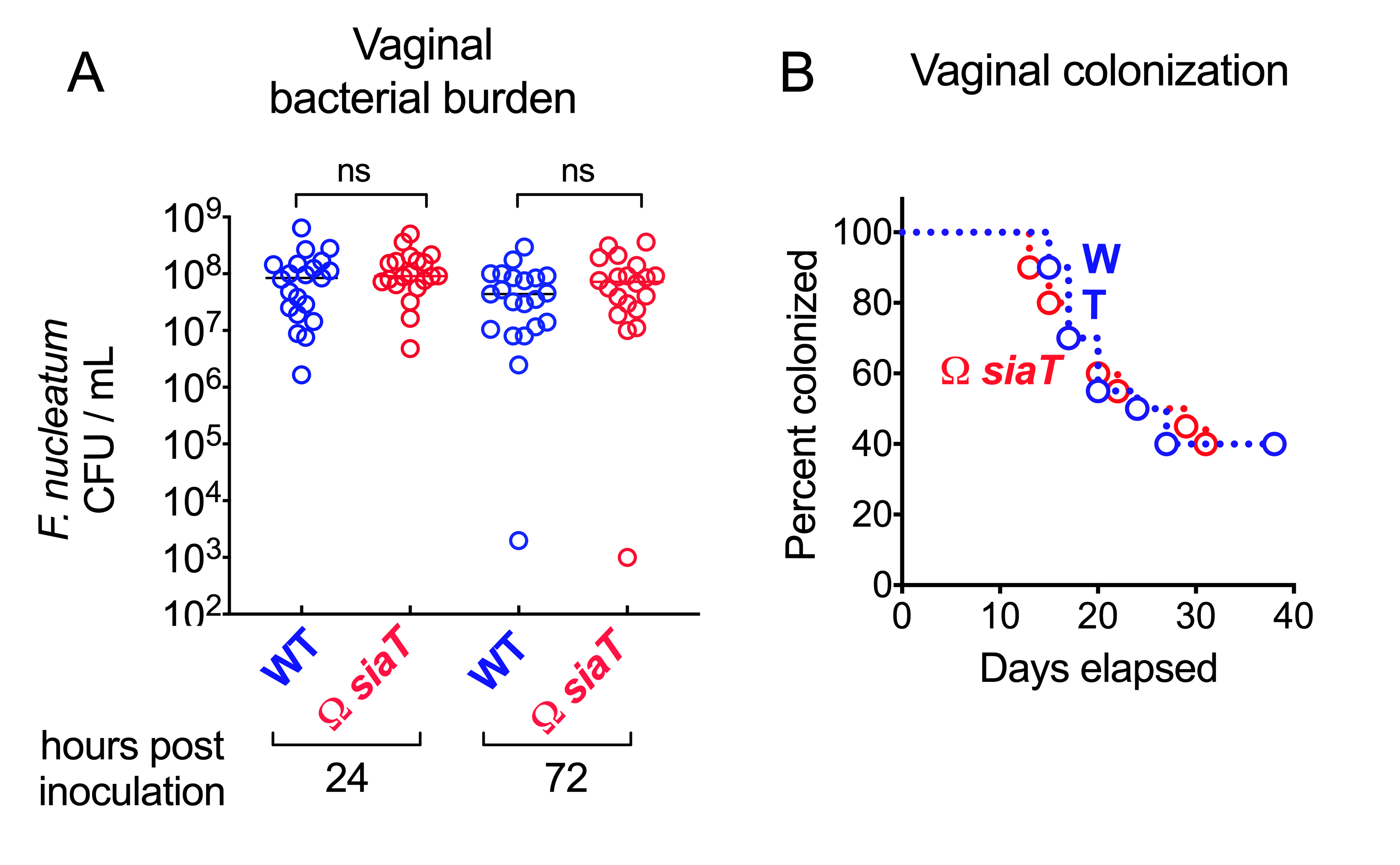

Supplement: S5 Fig — (A) F. nucleatum titers in vaginal wash collected at the indicated time points postinoculation. (B) Comparison of vaginal colonization with the WT versus ΩsiaT. Number of mice colonized in percent (y axis) was monitored on day 1 and every 2 days thereafter for 38 days (x axis). For Kaplan–Meier analysis, mice were considered cleared when no CFUs were detected in undiluted wash at 2 consecutive time points. Data are combined from 2 independent experiments. Each experiment had 10 mice per group. Statistical significance assessed by the Gehan–Breslow–Wilcoxon test revealed no significant difference in colonization by WT versus ΩsiaT in mice from Jackson. The underlying numerical data for this figure can be found in S1 Data. CFU, colony-forming unit; siaT, predicted sialic acid transporter; WT, wild type. (TIFF) [file pbio.3000788.s005.tiff]

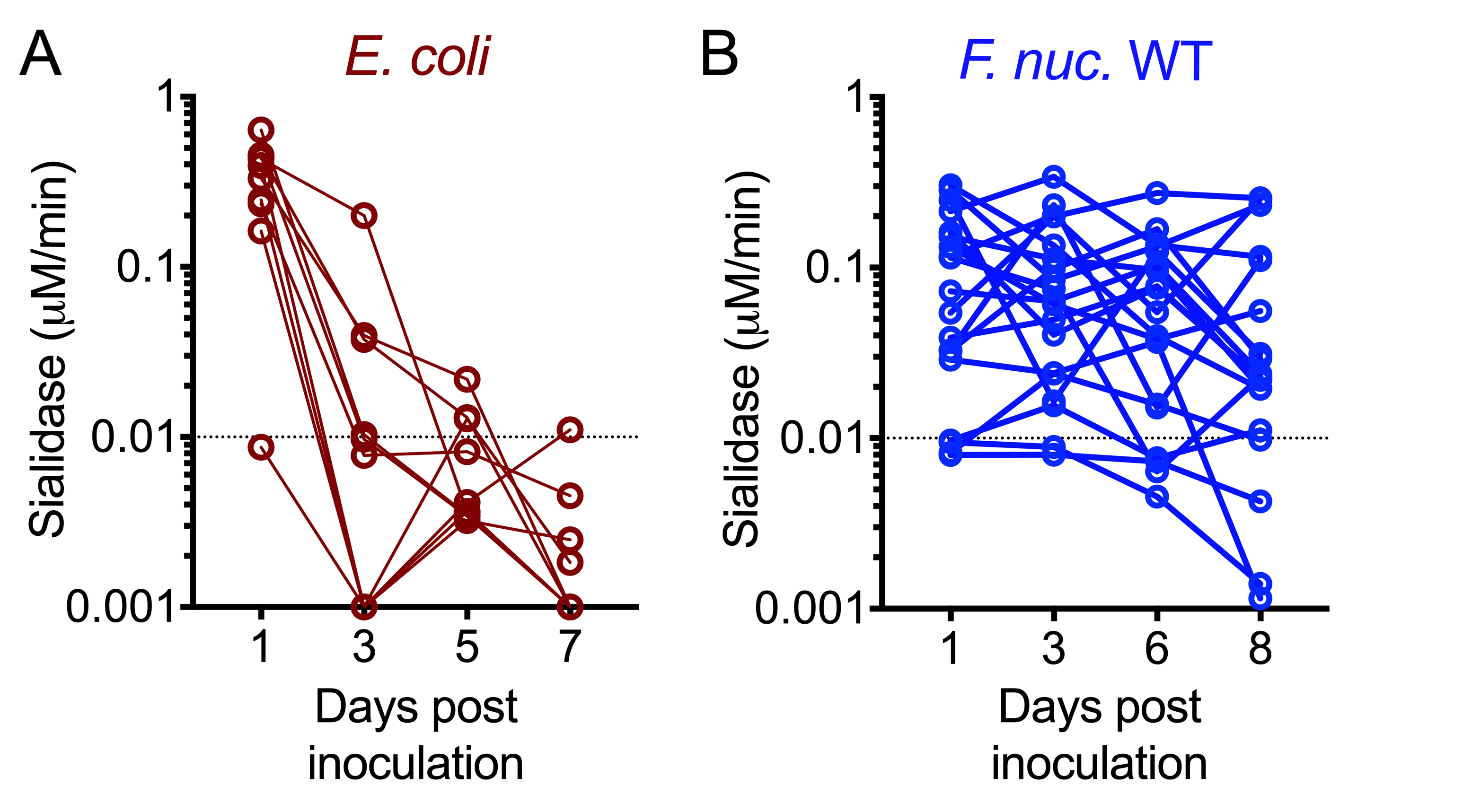

Supplement: S6 Fig — (A–B) Sialidase activity in vaginal washes from individual animals purchased from Envigo, estrogenized, and inoculated with E. coli (A) or F. nucleatum (B) from 1 to 8 dpi. The underlying numerical data for this figure can be found in S1 Data. dpi, days postinoculation. (TIFF) [file pbio.3000788.s006.tiff]

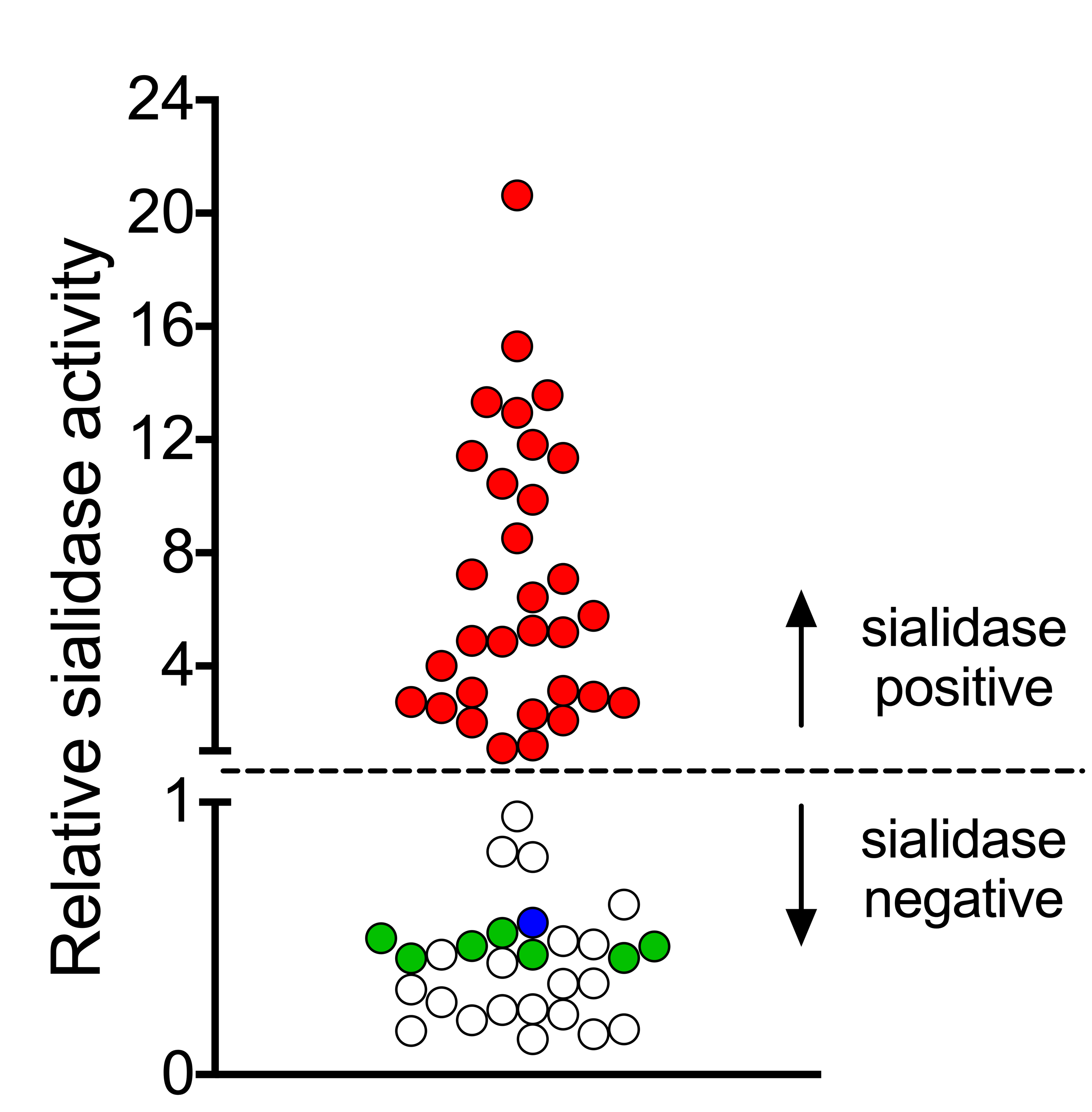

Supplement: S7 Fig — (A) Graph shows relative sialidase activity in vaginal swabs that were transported aerobically from clinic to the lab. Sialidase activity was measured in swab eluates (N = 58) using a 4MU-Sia assay. Samples with a relative sialidase activity >1.0 were considered to be sialidase-positive. Red = sialidase-positive specimens, green = blank swab controls, blue = buffer control. The underlying numerical data for this figure can be found in S1 Data. 4MU, 4-methylumbelliferone. (TIFF) [file pbio.3000788.s007.tiff]

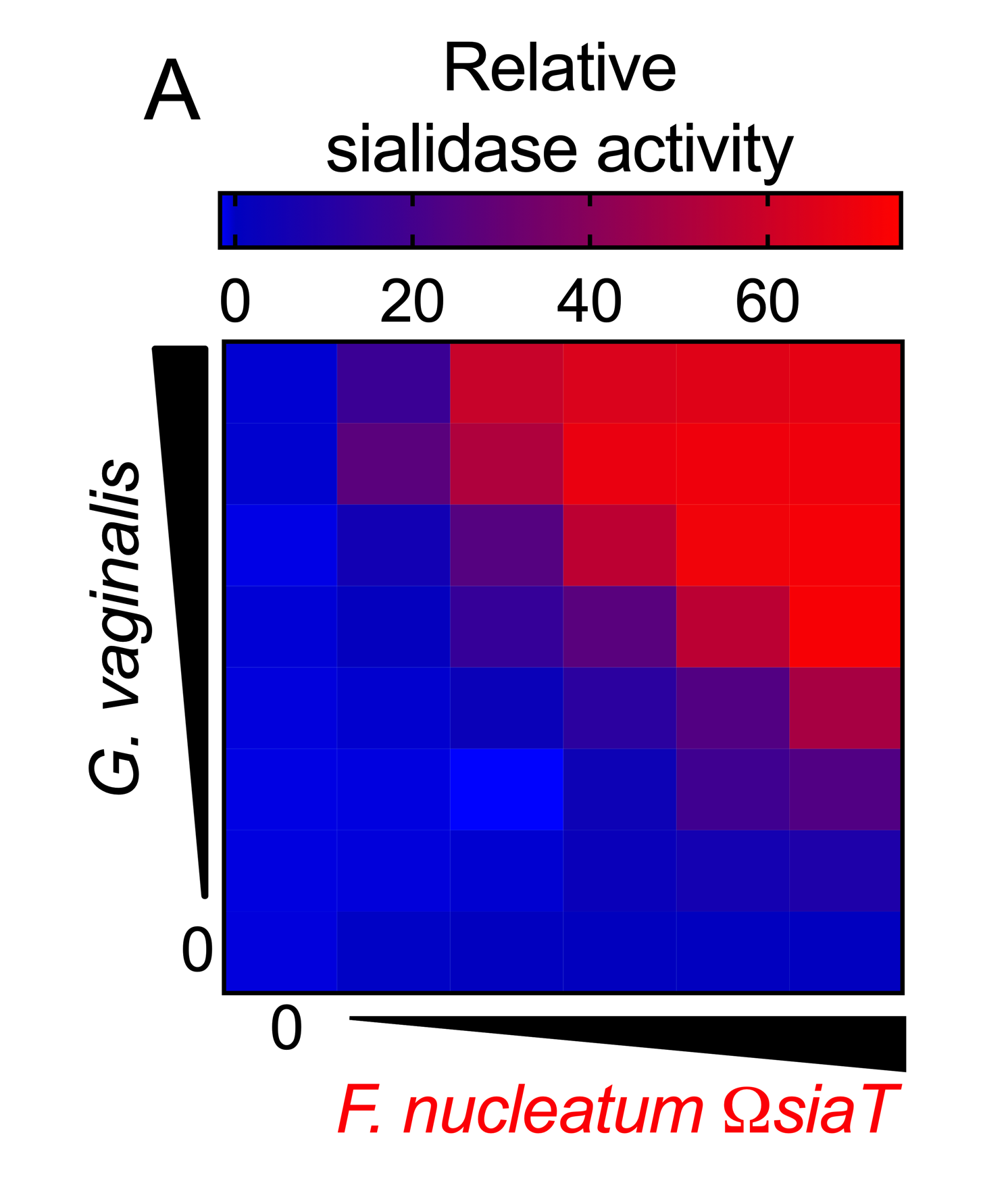

Supplement: S8 Fig — (A) Heat map shows relative sialidase activity in G. vaginalis (inoculum OD600 0.0–0.05) cultured overnight with F. nucleatum ΩsiaT (inoculum OD600 0.0–0.1) in supplemented Columbia media. Data shown are representative of 2 independent experiments. OD, optical density; siaT, predicted sialic acid transporter. (TIFF) [file pbio.3000788.s008.tiff]

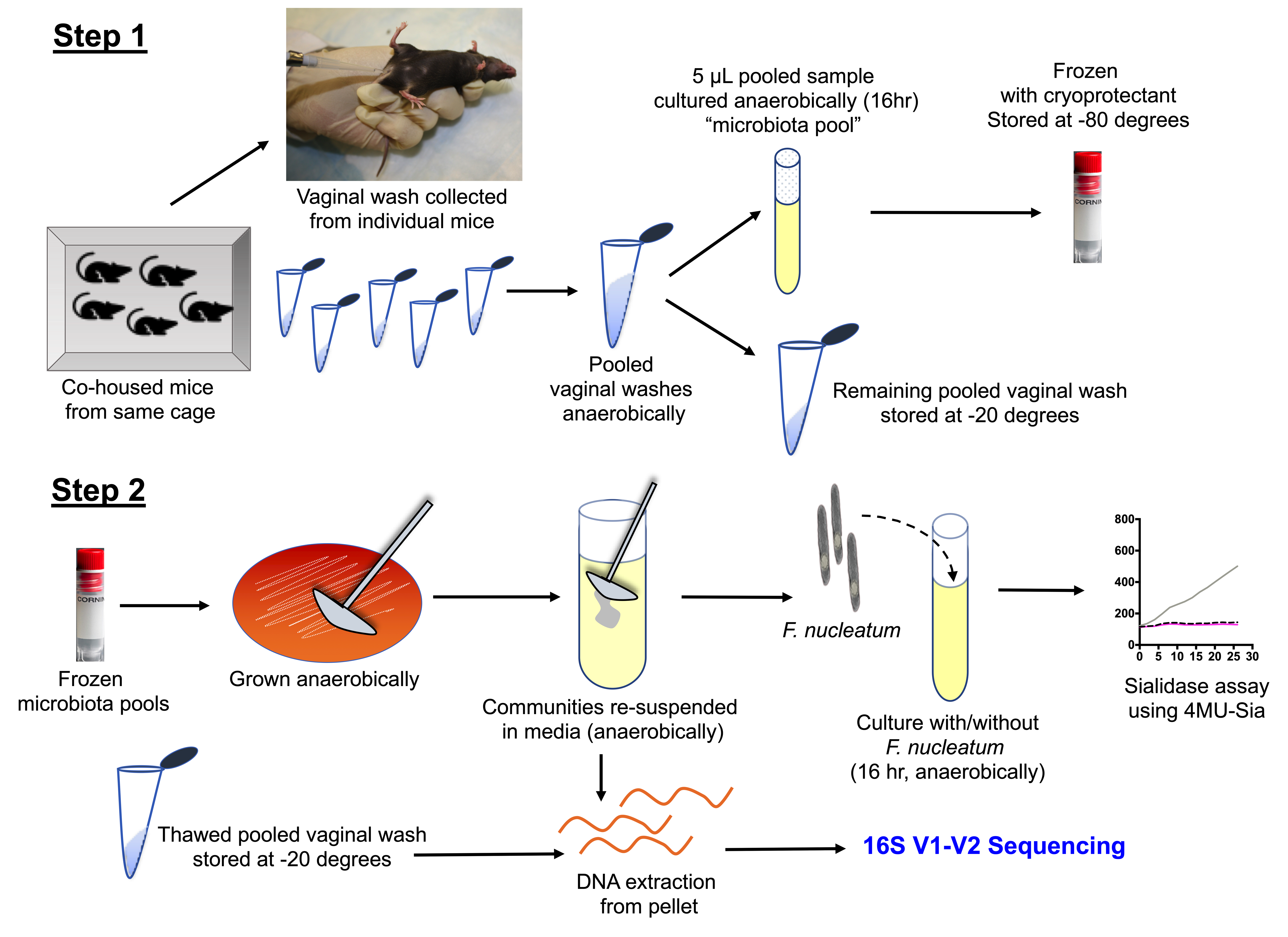

Supplement: S1 Schematic — Step 1: collection of mouse vaginal washes. Publication of this animal image was approved by IACUC. Washes were pooled from mice cohoused in the same cage. A portion of vaginal wash pools was cultured in Columbia broth (referred to as “microbiota pools”) to amplify the vaginal bacteria and frozen for subsequent use. Remaining pooled material was stored at −20°C. Step 2: on the day of the experiment, frozen microbiota pools were used to recover mouse vaginal bacteria by streaking out on supplemented Columbia blood plates in anaerobic chamber and incubating for 24 h at 37°C. Colonies from these plates were resuspended in liquid media either (a) for DNA extraction or (b) for coculture experiments with F. nucleatum. IACUC, Institutional Animal Care and Use Committee. (TIFF) [file pbio.3000788.s009.tiff]

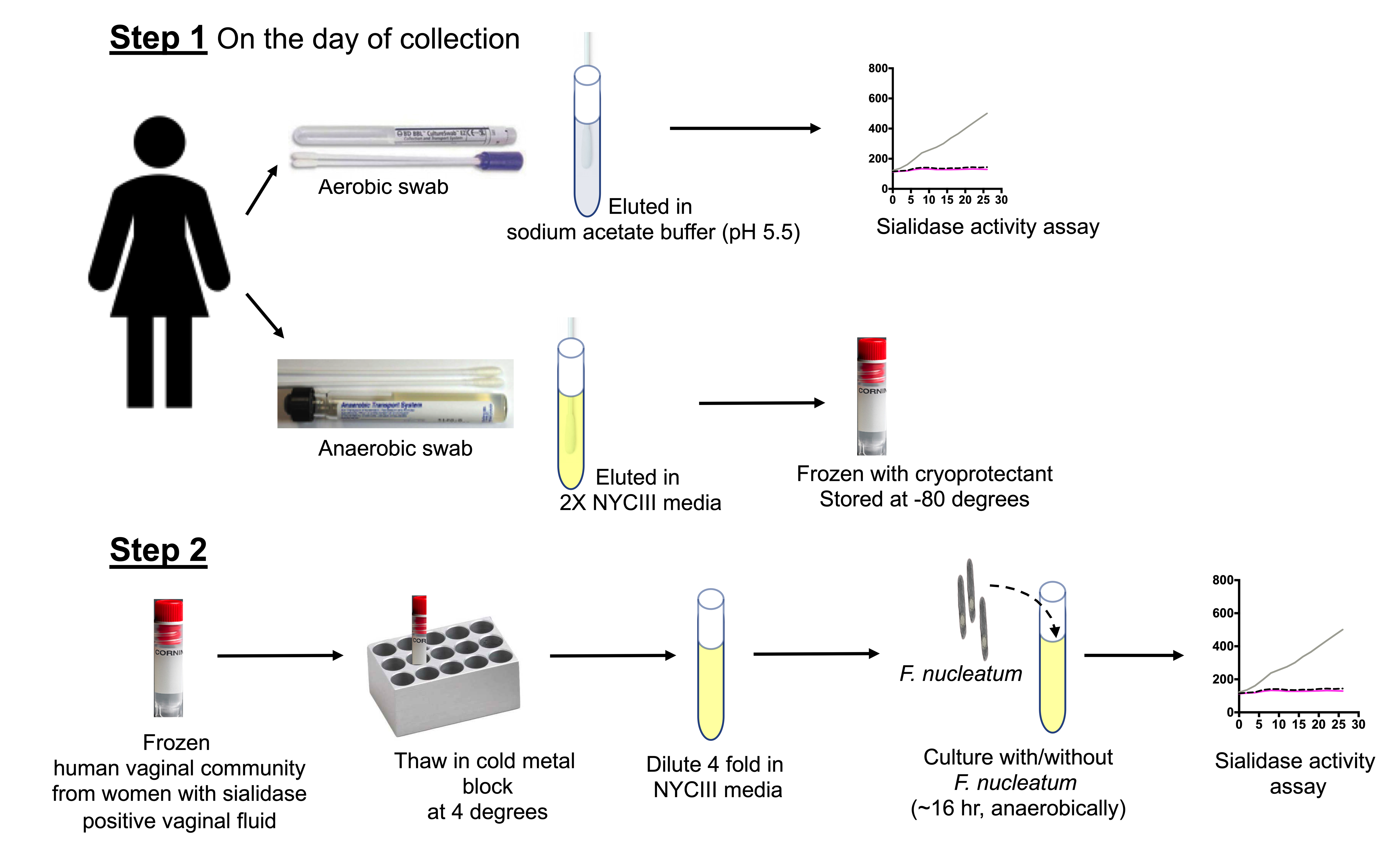

Supplement: S2 Schematic — Step 1: anaerobic and aerobic vaginal swabs were collected on the same day from each participant. Aerobic swabs were eluted in sodium acetate buffer (pH 5.5), and sialidase activity was checked in the swab eluates using fluorogenic 4MU-Neu5Ac substrate. Anaerobic swabs were eluted in 2× NYCIII media (in an anaerobic chamber) and the communities were “fresh frozen,” without any amplification/overnight culture, by mixing with cryoprotectant and storing at −80°C. Step 2: on the day of the experiment, fresh-frozen anaerobic vaginal communities, from women who had detectable sialidase activity in their aerobic swab eluates, were thawed at 4°C and diluted 4-fold in NYCIII media (in an anaerobic chamber). The diluted communities were used for coculture experiments with F. nucleatum. Neu5Ac, N-acetylneuraminic acid; 4MU, 4-methylumbelliferone. (TIFF) [file pbio.3000788.s010.tiff]
