# Supplementary material for: Glycan cross-feeding supports mutualism between Fusobacterium and the vaginal microbiota
Source: PLoS Biol. 2020 Aug 25;18(8):e3000788. doi: 10.1371/journal.pbio.3000788 (PMC7447053; doi:10.1371/journal.pbio.3000788)
Supplement: S1 Table — (PDF) [file pbio.3000788.s011.pdf]

S1 Table. Key bacterial strains, plasmids and primers

Name	Description	Source or reference
**Strains**		
E. coli MG1655	WT K12 strain	*E. coli* Genetic Stock Center
E. coli LSR4	MG1655 *ΔnanA*, sialate lyase mutant	[1]
E. coli Top10	Cloning strain	Invitrogen
F. nucleatum nucleatum ATCC25586		ATCC
F. nucleatum nucleatum ATCC23726		ATCC
F. nucleatum nucleatum ATCC23726 Sm^R	Spontaneous streptomycin-resistant mutant of ATCC23726	This study
F. nucleatum nucleatum ATCC23726 Sm^R Ω*siaT*	ATCC23726 Sm^R with plasmid disruption in *siaT* , sialic acid transporter mutant	This study
F. nucleatum polymorphum ATCC10953		ATCC
F. nucleatum vincentii JMP2A		[2]
F. nucleatum animalis FQG51A		[2]
F. nucleatum polymorphum JMSY1		Isolate provided by Justin Merritt
F. nucleatum JMH52		Isolate provided by Justin Merritt
*F. mortiferum ATCC*25557		ATCC
F. peridonticum SYJL4		[2]

Leptotrichia spp. JM6		Isolate provided by Justin Merritt
L. hofstadii JM4		Isolate provided by Justin Merritt
G. vaginalis JCP8151B	Human vaginal isolate	[3]
**Plasmids**		
pUC19	*E. coli* cloning vector	provided by Scott Hultgren
pJIR418	*C. perfringens* shuttle vector	[4]
pLR23	*catP* from pJIR418 fused with the pUC19 origin of replication--*Fusobacterium* suicide vector	This study
pLR25	pLR23 with 0.5 kb fragment from ATCC23726 *siaT*	This study
pTrc99A	*E. coli* expression vector	provided by Scott Hultgren
pLR7	MG1655 *nanA* in pTrc99A: *E. coli* sialate lyase complementation vector	This study
pLR10	ATCC23726 *nanA-his* in pTrc99A: *Fusobacterium nucleatum* sialate lyase complementation vector	This study
**Primers**		
pUC19 ori F Sac Kpn Nco	TTTTCCATGGTATAGGTACCTTATGAGCTCGT TCCACTGAGCGTCAG	This study
pUC19 ori R Bgl2	AAATAGATCTCGCAGGAAAGAACATGTGAGC	This study
catP F Bgl2	AAAAGATCTGAGCTAAAGAGGTCCCTAGC	This study
catP R Bam Pst Nco	AAAACCATGGATATCTGCAGATATGGATCCAA G GTCTTTGTACTAACCTGTGG	This study
23726 nan F Nco	AAAACCATGGCCTCTTATTTGGAGTGAAGAGC	This study

23726 nan R Sac	AAAAGAGCTCAAGTAAGGACCAACCAACAG	This study
23726 nan test F1	GAGTGGCTAGGAGGAAGTC	This study
pLR24 5’ F	GTTTGCAAGCAGCAGATTACG	This study
MG1655 nanA F Nco	AAAACCATGGCAACGAATTTACGTGG	This study
MG1655 nanA R Pst	AAAACTGCAGGAAAATTGTAGGGTACAGATGC G	This study
Fuso nanA F Nco	AAAACCATGGCAAAAGGGATATATTCAGCATT GATG	This study
Fuso nanA his R Bam	TTTTGGATCCTTAGTGGTGGTGGTGGTGGTG ATTTT TTAAAAATTTTTTATGAAGTTCTTTTGC	This study
EFG95902 RT Fp	TGGAGCAGAACCTCAATCATAC	mutarotase<br>This study
EFG95902 RT Rp	CAAGGTGCATCAAATGGTACTTC	mutarotase<br>This study
EFG95903 RT Fp	TATGTGGGAACTGACTACCAAA	ccpA<br>This study
EFG95903 RT Rp	GCCCTGTCTAAGAAACCATTTAG	ccpA<br>This study
EFG95904 RT Fp	GGTGTTATGACAACTTCTGCTTT	siaP<br>This study
EFG95904 RT Rp	GCAAATACTTCTGCTGCCTTG	siaP<br>This study
EFG95905 RT Fp	AGTGGCTAGGAGGAAGTCTATT	siaT (A) Fwd primer<br>This study
EFG95905 RT Rp	CTTCACTCCAAATAAGAGGGCTAT	siaT (A) Rev primer<br>This study
SiaT insert RT Fp	CTGTTGGTTGGTCCTTACTTGT	siaT (B) Fwd Primer<br>This study

SiaT insert RT Rp	TCCAGTAAGTATGAAGAATGGAACAC	siaT (B) Rev Primer<br>This study
EFG95906 RT Fp	GATGTGAACTGTGTTGCTCTTG	Kinase<br>This study
EFG95906 RT Rp	CCTCCACCTATACCTGTTCCTA	Kinase<br>This study
EFG95907 RT Fp	TGATGGTTCCATACAATGAAGATG	nanA<br>This study
EFG95907 RT Rp	CCCAGTACTTCCACCTACATATAA	nanA<br>This study
EFG95908 RT Fp	CCAGTGATAGCTGAGGGAAATA	nanE<br>This study
EFG95908 RT Rp	GGTCTAGTTATTGCTCCACCA	nanE<br>This study
EFG95909 RT Fp	GTGCTTCAACAGCTACTTTATCAG	Transporter<br>This study
EFG95909 RT Rp	TAGCATTCCTTCTCCAACTTCTT	Transporter<br>This study
Fn 16S rRNA RT Fp	CTTAGGAATGAGACAGAGATG	[5]
Fn 16S rRNA RT Rp	TGATGGTAACATACGAAAGG	[5]
Gvag_tuf_AS3	RCGCAAACCAACRATCTCAACTGG	[6]
Gvag_tuf_S4	TCCCAACCCCAACTCACGATCTT	[6]

References

1. Robinson LS, Lewis WG, Lewis AL. The sialate O-acetylesterase EstA from gut *Bacteroidetes* species enables sialidase-mediated cross-species foraging of 9-O-acetylated sialoglycans. J Biol Chem. 2017;292(28):11861-72. Epub 2017/05/21. doi: 10.1074/jbc.M116.769232. PubMed PMID: 28526748; PubMed Central PMCID: PMCPMC5512079.

2. Yoneda S, Loeser B, Feng J, Dmytryk J, Qi F, Merritt J. Ubiquitous sialometabolism present among oral fusobacteria. PloS one. 2014;9(6):e99263. doi: 10.1371/journal.pone.0099263. PubMed PMID: 24896093; PubMed Central PMCID: PMCPMC4045943.

3. Gilbert NM, Lewis WG, Lewis AL. Clinical features of bacterial vaginosis in a murine model of vaginal infection with *Gardnerella vaginalis*. PLoS One. 2013;8(3):e59539. Epub 2013/03/26. doi: 10.1371/journal.pone.0059539. PubMed PMID: 23527214; PubMed Central PMCID: PMC3602284.

4. Sloan J, Warner TA, Scott PT, Bannam TL, Berryman DI, Rood JI. Construction of a sequenced *Clostridium perfringens-Escherichia coli* shuttle plasmid. Plasmid. 1992;27(3):207-19. Epub 1992/05/01. PubMed PMID: 1513878.

5. Rubinstein MR, Wang X, Liu W, Hao Y, Cai G, Han YW. *Fusobacterium nucleatum* promotes colorectal carcinogenesis by modulating E-cadherin/beta-catenin signaling via its FadA adhesin. Cell Host Microbe. 2013;14(2):195-206. Epub 2013/08/21. doi: 10.1016/j.chom.2013.07.012. PubMed PMID: 23954158; PubMed Central PMCID: PMCPMC3770529.

6. Balashov SV, Mordechai E, Adelson ME, Gygax SE. Identification, quantification and subtyping of *Gardnerella vaginalis* in noncultured clinical vaginal samples by quantitative PCR. J Med Microbiol. 2014;63(Pt 2):162-75. Epub 2013/11/10. doi: 10.1099/jmm.0.066407-0. PubMed PMID: 24200640.
