# Supplementary material for: Glycan cross-feeding supports mutualism between Fusobacterium and the vaginal microbiota
Source: PLoS Biol. 2020 Aug 25;18(8):e3000788. doi: 10.1371/journal.pbio.3000788 (PMC7447053; doi:10.1371/journal.pbio.3000788)
Supplement: S2 Table — ATCC, American Type Culture Collection. (PDF) [file pbio.3000788.s012.pdf]

S2 Table. Genetic organization of the putative sialic acid catabolic gene cluster in *F. nucleatum* strain ATCC 23726.

ORF start	ORF end	Putative function of encoded protein	Accession number (Genbank)
61776	62924	*N*-acetylneuraminic acid mutarotase	EFG95902
62942	63943	LacI family regulator *ccpA*	EFG95903
63963	64946	sialic acid binding protein *siaP*	EFG95904
64970	66823	acetylneuraminate ABC transporter *siaT*	EFG95905
66826	67701	*N*-acetylmannosamine kinase	EFG95906
67723	68595	*N*-acetylneuraminate lyase (sialate lyase) *nanA*	EFG95907
68610	69284	*N*-acetylmannosamine-6-phosphate epimerase *nanE*	EFG95908
69443	70330	membrane transporter	EFG95909
