# Supplementary material for: Glycan cross-feeding supports mutualism between Fusobacterium and the vaginal microbiota
Source: PLoS Biol. 2020 Aug 25;18(8):e3000788. doi: 10.1371/journal.pbio.3000788 (PMC7447053; doi:10.1371/journal.pbio.3000788)
Supplement: Attachment — Submitted filename: Responses to Reviewers 05122020_AL.docx [file pbio.3000788.s015.docx]

**Responses to Reviewers for**

Glycan cross-feeding supports mutualism

between *Fusobacterium* and the vaginal microbiota

Kavita Agarwal,^1,2^ Lloyd S. Robinson,^1,2^ Somya Aggarwal,^1,2^ Lynne R. Foster,^1,2^ Ariel Hernandez-Leyva,^2,6^ Hueylie Lin,^1,2^ Brett A.Tortelli,^2,5^ Valerie P. O’Brien,^1,2^ Liza Miller^1,2^, Andrew L. Kau,^2,6^ Hilary Reno,^7^ Nicole M. Gilbert,^2,3,4^ Warren G. Lewis,^1,2,*^ Amanda L. Lewis^1,2,3,^*

**Affiliations:**

^1^Department of Molecular Microbiology, Washington University School of Medicine, St. Louis, MO 63110, United States of America.

^2^Center for Women's Infectious Disease Research, Washington University School of Medicine, St. Louis, MO 63110, United States of America.

^3^Department of Obstetrics and Gynecology, Washington University School of Medicine, St. Louis, MO 63110, United States of America.

^4^Center for Reproductive Health Sciences, Washington University School of Medicine, St. Louis, MO 63110, United States of America.

^5^Department of Genetics, Washington University School of Medicine, St. Louis, MO 63110, United States of America.

^6^Division of Allergy and Immunology, Department of Medicine, Washington University School of Medicine, St. Louis, MO 63110, United States of America.

^7^Division of Infectious Disease, Department of Medicine, Washington University School of Medicine, St. Louis, MO 63110, United States of America.

*Correspondence: allewis@wustl.edu, mandylew@gmail.com (A.L.L.), WarrenGLewisPhD@gmail.com (W.G.L.)

**Authors:** We appreciate the reviewer’s positive feedback on the manuscript, for example, “manuscript presents some very interesting and novel insights into the interplay between sialic acid catabolism and the vaginal microbiota” and “present compelling evidence that certain strains of F. nucleatum can grow on free sialic acid and are thereby provided an advantage in environments where sialidases are present”. We have used the many constructive comments and suggestions to improve the manuscript, including the addition of data and by revising the manuscript to clarify and discuss the issues raised. We thank the reviewers for taking the time to perform such a thorough and constructive review of our paper. Author responses to the Reviewers are shown in blue text to set them apart from the Reviewer comments, shown in black text.

Note for Reviewers: In the responses, the line numbers refer to the document provided in tracked changes mode (marked as “TRACKED” in the file name).

**Point by Point Responses to Reviewer Comments**

**Reviewer 1:**

In their manuscript “Glycan cross-feeding supports mutualism between Fusobacterium and the vaginal microbiota”, Agarwal et al set out to validate the hypothesis that the increased risk for pathogenic colonization observed in bacterial vaginosis patients is due (at least in part) to the increased presence of sialidases. Specifically, the authors look at the pathogen Fusobacterium nucleatum, which had been predicted to be able to catabolize free sialic-acid but does not possess sialidases. They generate a knockout (siaT) that is lacking key metabolic genes that block the ability of F. nucleatum to catabolize and grow in the presence of free sialic acid, which is then used as a control for the next experiments to distinguish between effects caused by the presence versus catabolic activity of F. nucleatum. Overall Agarwal et al present compelling evidence that certain strains of F.nucleatum can grow on free sialic acid and are thereby provided an advantage in environments where sialidases are present. However, there are some points in the manuscript that need clarification as detailed below.

**Authors:** We thank Reviewer 1 for indicating that the arguments supporting our overall conclusion is compelling and for suggesting ways we can further improve support for our overall model.

Major Comments

**Reviewer 1: 1.1**-     The murine in vivo results show that only the WT, not siaT mutant, increase sialidase activity over time (Fig 5B+C). First, it is not clear if the siaT mutant increases sialidase activity compared to no infection control (albeit less than WT). Second, the murine ex vivo results show that both WT and siaT mutants increase sialidase activity in the vaginal microbial community. What accounts for this discrepancy between the in vivo and ex vivo data? This raises major concerns how translational these findings are.

**Author Response:** We clarify that our murine *in vivo* results show that sialidase activity decreases over time once animals are received in our facility, but persists at higher levels in mice that receive WT *Fusobacterium* [compared to mock treatment (shown in Fig 5A) or the siaT mutant (shown in Fig 5C)]. We believe the discrepancy in the *ex vivo* and *in vivo* results is explained by a dose dependent effect of *F. nucleatum* on sialidase activity (shown in Fig 6D) in mouse vaginal communities. For *ex vivo* studies, we have now clarified in the methods that equal inocula (OD normalized) of Fuso WT and *siaT* were added to each community and experiments were done in a medium where both strains grow equally well and reach the same final titers (shown in Fig 6C). However, variation of the starting inoculum ex vivo resulted in a dose-dependent effect (Fig 6D). These data suggest that the difference in sialidase activity *in vivo*, where WT colonization is more persistent than siaT, is likely due to the difference in *F. nucleatum* titers in the groups infected with WT and siaT. We try to explain this in the manuscript (lines 363- 366) – “These data suggest that in mice, the divergence of sialidase activity in groups receiving WT versus Ω*siaT* arising after 6 days (**Fig 5C**) resulted from different ‘doses’ of *F. nucleatum* (arising as of day 3, **Fig 4C** and S4 Fig), rather than a specific feedback loop involving sialic acid catabolism.” We have now provided the CFU data for 6 and 8 dpi in Envigo mice as supporting information, and explain in the results that the *siaT* titers are significantly lower than WT at these time points.

**Reviewer 1: 1.2** -     In the human vaginal swab ex vivo data, only the WT F. nucleatum strain is used, I assume that is because there was a limited amount of sample but it would be interesting to see if the mutant results here reflect the in vivo or ex vivo results from the murine experiments.  Additional experiments would help to address this question.

**Author Response:** We thank the reviewer for this suggestion and agree that this is an interesting future direction. We will try to design future studies with appropriate power calculations and available sample volumes to ask this question.

Minor Comments
**Reviewer 1: 1.3** -     Figure 6;
     **1.3a** Fig6; as written the experiment is not clear; frozen vaginal microbial communities were grown overnight and then mixed with an F. nucleatum strain to test for effect – for how long were they grown again? It took me a while to find this in Fig S1 (16hrs), I would recommend mentioning this in the results for improved clarity.

**Author Response:** We thank the reviewer for catching this and agree that this is key information that should be readily accessible. We have now added this information in the legend for Fig 6, as well as to the methods and results sections for clarity.

**Reviewer 1:**  **1.3b** Fig 6C shows the final F. nucleatum CFU count after incubation with the vaginal pools, but how much F. nucleatum was put in to start? Is this concentration-dependent? It would be nice to see that the amount added was similar between both strains at the beginning as well.

**Author Response:** We thank the reviewer for noticing this omission. We now more clearly explain in the results and methods that the inoculum of WT and mutant strains were normalized at the outset in these experiments.

**Reviewer 1:**     **1.3c** The CFU input should be listed in Fig 6D (at a minimum in the legend if not on the figure) since right now it just shows qualitative increased input with triangles – is the increasing input different by 2-fold? 10-fold? 0.1-fold?

**Author Response:** We thank the reviewer for this suggestion. We have now added this information in Fig 6 and the methods for clarity. Communities were cultured with either *no F. nucleatum* or OD normalized inocula of WT and *siaT* strains with OD600 = 0.10 or 4 -fold lower OD600 = 0.025.

**Reviewer 1: 1.4** Fig S4; Even though there is no difference between WT and KO colonization in Jackson mice, when comparing numbers to Envigo mice in Fig 4 it appears that the KO is better at colonizing in Jackson mice rather than the WT being worse (as I would expect since Jackson mice have low sialidase activity) – maybe colonization in these mice is driven by different factors? I think it would be worthwhile to comment on this at least in the supplementary info.

**Author Response:** We agree with the Reviewer. Please find our comments on this in the discussion in lines 508-515.

**Reviewer 1: 1.5** -     Lines 260-270; the way this is currently written it sounds like sialidase activity was somehow determined from the extracted DNA – clarifying that 16S rRNA gene amplicon sequencing was performed and in parallel that isolated colonies were screened for sialidase activity will improve the readability of this section.

**Author Response:** We appreciate this suggestion and have edited the text in this result section to describe the experimental approach more clearly.

**Reviewer 1: 1.6** -     Line 273; ‘somewhat different’ is not very descriptive, from Sup Fig3D it appears as though the communities are quite distinct, a PERMANOVA analysis would help strengthen this statement in the text.

**Author Response:** We thank the Reviewer for this suggestion and agree that “somewhat different” was not that descriptive. We clarify that our rationale for doing this experiment was “to investigate whether sialidase activity in vaginal washes was of bacterial origin.” We have now revised this section to better streamline and focus the content toward this intent. Along these lines, we have removed the sentence stating that Envigo mice had a “somewhat different” vaginal microbiota compared to Jackson mice. Instead, we now focus on the specific experimental findings that “strongly support the interpretation that C57BL/6 mice from Envigo exhibit vaginal sialidase activity arising from the endogenous microbiota.”

We will make these raw data available to other researchers should they have other hypotheses to test using PERMANOVA or other analyses.

**Reviewer 1: 1.7** -     Line289; clarify to lower CFU titers.

**Author Response:** We have clarified this section, which now reads, “As early as 72 hours post inoculation (hpi), and later at 6 days post inoculation (dpi) upto 8 dpi, Ω*siaT* had significantly lower titers than WT in Envigo mice (**Fig. 4C** and S4 Fig)”.

**Reviewer 1: 1.8**  Fig5B; difficult to tell which groups the stars are signifying significance for, use bars like in 5C.

**Author Response:** We have updated Fig 5B as suggested by the reviewer in the revised manuscript.

**Reviewer 1: 1.8** Fig S5; E.coli misspelled in legend (E.colii).

**Author Response:**We have made this correction in the caption for S6 Fig, which was earlier Fig S5, in the revised manuscript.


**Reviewer 2:**

**Reviewer 2:** The manuscript reports the characterisation of sialic acid consumption by Fusobacterium nucleatum strains and the impact on sialic acid cross-feeding on F. nucleatum colonisation of the vaginal niche.  Specific outputs include the characterisation of NanA (sialic acid lyase) and NanT (sialic acid transporter) as critical components of F. nucleatum sialic acid metabolism in vitro, the ability of F. nucleatum to benefit from a sialidase-rich vaginal microbiota and the impact of F. nucleatum on G. vaginalis. The methodology is sound, and the manuscript presents some very interesting and novel insights into the interplay between sialic acid catabolism and the vaginal microbiota.

Below some comments and suggestions to clarify some aspects of the manuscript.

**Authors:** We thank Reviewer 2 for their insightful comments. We appreciate the constructive criticism and have added text and data to the paper to improve clarity and provide additional support for our model.

**Reviewer 2:**

**2.1** In the first set of experiments,  **[note: this phrase is applied in the sub-comments below, in brackets]**

**2.1a** The authors compared the Neu5Ac levels of F. nucleatum strains grown on (rich?) growth medium as compared to un-inoculated medium but don't provide information (in Results or in Fig legend) on the composition of the medium and whether it was supplemented with Neu5Ac (using the semi-defined medium introduced on p. 8. This information should be provided.

**Author Response:** We have made efforts to further clarify this issue in the legend to Figure 1, which now reads in part, “Subspecies of *F. nucleatum* are indicated in blue text. All fusobacteria were grown in (rich) supplemented Columbia medium with free sialic acid added (Neu5Ac, 100 µM) - medium that was also used as un-inoculated control.”

**Reviewer 2: 2.1b [**In the first set of experiments,] the analysis uses un-inoculated medium as a control, a more informative control would be to use medium with no Neu5Ac as carbohydrate source.

**Author Response:** Here we are examining the consumption of sialic acid by *Fusobacterium*. Later in the paper we also evaluate the influence of added Neu5Ac on growth (see Fig 2E). We agree with the Reviewer that in the setting of growth, the requested control is key to interpreting the results. Here (Fig 1A) we are evaluating the extent to which the indicated strains consume the added free Neu5Ac. To evaluate this, we needed to show the starting content of Neu5Ac in a setting where there are no bacteria present to consume it.

**Reviewer 2: 2.1c** **[**In the first set of experiments,] the authors should also include in the Results section how the Neu5Ac concentrations were determined (p. 5), and how they measured free versus bound sialic acid (p.11).

**Author Response:** We acknowledge that this was not well-explained and thank the Reviewer for pointing this out. We have now added a brief explanation in the results in lines 147-149, which reads: “The amount of remaining sialic acid was measured by fluorescent derivatization and HPLC analysis by comparison to synthetic Neu5Ac standards processed in parallel.” We have also added additional explanation to the text to clarify how free and bound sialic acid were determined: “As described more fully in the methods, derivatization without prior acid hydrolysis measures free Neu5Ac, while derivatization following acid hydrolysis, which releases bound Neu5Ac from sialoglycans, measures the total Neu5Ac (Bound Neu5Ac = Total Neu5Ac - Free Neu5Ac).” on p. 8 line 214-217

**Reviewer 2: 2.1d** The authors demonstrated that in vitro F. nucleatum is not able to utilise a synthetic sialylated substrate and that adding exogenous sialidase from Arthobacter ureafaciens enabled growth of F. nucleatum on this substrate. The interpretation of the data is based on growth measurements and supporting data such as measuring the Neu5Ac in the medium and/or the sialidase activity during growth would be needed to strengthen the interpretation.

**Author Response:** We agree with the Reviewer that Neu5Ac measurements support our interpretation that *F. nucleatum* is not able to utilize a sialylated substrate (in the bound form) except in the presence of added sialidase. The supporting Neu5Ac measurements are provided in Fig 3D.

**Reviewer 2: 2.1e** Also, the authors may comment why G. vaginalis and not Arthobacter ureafaciens was used as a control in the first growth experiment (L.213) or conversely why the sialidase from G. vaginalis was not used in the following experiment (from L.218).

**Author Response:** Please see response to 2.1f below.

**Reviewer 2: 2.1f** The authors should also provide information on the linkage of the substrate as differences in sialidase specificity of the bacteria tested will need to be considered when interpreting the results.

**Author Response:** We interpreted that comments 2.1e and 2.1f are interrelated and will address them together. Here, our intent was to illustrate that the *F. nucleatum* strain (ATCC23726), used as the main WT strain for study in this manuscript did not produce sialidase activity. The *G. vaginalis* sialidase activity assayed here (Fig 3A) was derived from cultured *G. vaginalis* (JCP 8151B, a known sialidase producing vaginal strain). Later experiments required more precision and specificity and we chose to use a more purified sialidase available from a commercial source (*Arthrobacter*). Both *Gardnerella* and *Arthrobacter* are *Actinobacteria* and we note that sialidases from both organisms have been studied and both cleave a wide spectrum of sialic acid substrates, including 2-3 and 2-6-linked sialic acids. We have added the linkage information and a statement to address this point in the results in lines 222-224.

**Reviewer 2: 2.1g** Did the authors try and co-culture a sialidase-producing bacteria from the vaginal niche (e.g. G.vaginalis, Arthobacter ureafaciens or Enterococcus casseliflavus/gallinarum) with F. nucleatum on sialylated substrate?

**Author Response:** This is an excellent suggestion that we hope to accomplish in future studies. We have been able to devise low nutrient conditions for *F. nucleatum* (alone) as shown in Figure 3C. However, the optimization of low nutrient conditions for co-culture has been a major challenge, despite many attempts.

**Reviewer 2: 2.2** With regards to the mouse work, the authors demonstrate a link between vaginal microbial sialidase activity and F. nucleatum colonisation. **It is suprising that the authors did not measure the level of free Neu5Ac in the vaginal washes of the mice under study so to link the increase in F. nucleatum with increased availability of free Neu5Ac in mice displaying high sialidase activity. Did/could the authors carry out these investigations? These data would need to be included to be consistent with the title of the manuscript that glycan cross-feeding supports mutualism between Fusobacterium and the vaginal microbiota.**

**Author Response:**

We agree with the Reviewer that Neu5Ac measurements would add value to the mouse work and we did carry out some investigations (pre-coronavirus) to try to address this question (discussed below). It is reasonable to hypothesize that like sialidase, free sialic acid levels might also be linked with *F. nucleatum* colonization. However, there are attributes of our system that may limit our conclusions about free sialic acid as a reflection of overall sialic acid availability. First, although higher sialidase activity in mice colonized by WT (vs *siaT)* could theoretically create more free sialic acid in this group, WT will also consume the free sialic acid (while siaT cannot). Members of the endogenous mouse microbiome might also participate in sialic acid catabolism in this system. These opposing forces (sialidase liberating sia/microbes consuming sia) might limit our conclusions about free sialic acid levels as an indicator of overall sialic acid availability. On the other hand, we suggest that levels of *total* sialic acid might better reflect the cumulative influence of sialic acid release and catabolism in the system. Using this metric, we might expect higher *F. nucleatum* might be associated with lower levels (depletion) of total sialic acid content. According to this logic, if our cross feeding hypothesis is true and sialic acid is being liberated by sialidase producers and consumed by WT *F. nucleatum* *in vivo*, we might expect to see lower levels of total sialic acid in mice colonized by WT compared to mutant (*siaT)*.

Prior to the coronavirus-related shutdown of our lab, we performed both free and total sialic acid measurements for vaginal wash material that was available from a few animals at 8 dpi . At this time point the differences in both *F. nucleatum* titers (WT>siaT, S4 Fig) and sialidase activity (WT>SiaT, Fig 5C) were evident. One of the limitations of the mouse system is that only a small volume of vaginal wash material was available for sialic acid measurements, due to the required volumes for other downstream analyses (e.g. sialidase, cfu etc). Only a few microliters were available from each wash from N=18 mice (there was not enough material to do free and total measurements on samples from the same animals). **We now provide this data as supporting information (S4 Fig) in the manuscript** and note in the results section that, “Sufficient vaginal wash material was available for sialic acid measurement at 8 dpi from some of the mice in each experimental group. Free sialic acid levels were similar between WT and Ω*siaT* inoculated mice. However, total sialic levels were lower in mice colonized with WT compared to Ω*siaT* inoculated mice (S4B Fig). The latter comparison was underpowered with the number of samples available and did not reach statistical significance (P=0.19). The lower levels of total sialic acid in mice with *F. nucleatum WT* are consistent with a depletion of sialic acid, an observation also seen in women with BV.”

Mutualism is defined as a relationship where both sides benefit from each other. We agree with the reviewer that our data illustrate a link between vaginal microbial sialidase activity and *F. nucleatum* colonization (CFU). Our data show that this relationship does not require sialic acid foraging per se (as tested in vitro), but rather suggests that sialic acid cross-feeding supports *F. nucleatum* in persisting longer and at higher titers compared to the *siaT* mutant (Figure 4 and 5), thereby providing more benefit to the sialidase producers in the community under these conditions, as evident by higher sialidase activity in these group of mice at 6 and 8 dpi (Figure 5D). We acknowledge that the physiological complexity of the mouse model limits our conclusions somewhat *in vivo*. Nevertheless, we hope the reviewer will agree that together with the additional evidence provided using independent experimental strategies *in vitro*, our comparison of WT versus mutant in the mouse model provides strong support that each of the entities in the studied relationship benefit from each other and that the mutual relationship relies at least in part on sialic acid cross feeding. If the Reviewer still feels that the title needs tweaking to better reflect the overall conclusions from the full dataset, we are open to additional comments/suggestions and/or the opportunity to provide alternative title options.

**Reviewer 2: 2.3** The last part of the study revealing an increase in microbial sialidase activity of mouse or human vaginal communities in the presence of F. nucleatum is interesting but, is more of an observation, and more work would be needed to strengthen this part of the work, which may be outside the scope of this manuscript. I would therefore suggest removing these last two sections from the manuscript as without further supporting data, the possible explanations provided are too speculative at this stage.

**Author Response:** We agree with the reviewer that the observed increase in sialidase activity of mouse and human vaginal communities in presence of *F. nucleatum* is interesting and further studies will be important to understand such interactions among vaginal microbes. We also discuss the future directions and limitations of this work (lines 548-555). We argue that these findings are required to provide evidence of mutualism between *F. nucleatum* and vaginal microbiota (and support the overall conclusion of the manuscript). Although we do not yet fully understand the molecular basis of the benefit provided by *F. nucleatum* to sialidase-producing vaginal bacteria, we feel it would be abrupt and unsatisfying to end the manuscript with mouse studies that suggest mutualism, without testing whether the benefit to sialidase-producing bacteria also applies to microbes from the human vaginal microbiota. We hope the reviewer will agree that in spite of the need for future study, the final two figures are needed to convey the potential significance of the observed relationship in vaginal communities from women.

Minor comments.
**Reviewer 2: 2.4** The sentences L. 127 and L211 about the bioinformatics analysis of F. nucleatum 'proteomes' are ambiguous, as this analysis is based on genome screening not on proteomics data.

**Author Response:**  We thank the reviewer for noticing this ambiguity. We clarify in the methods that the bioinformatics analysis was done using an amino acid sequence of *E. coli* NanA for identification of protein homologs in *F. nucleatum*. We have now revised the scale bar in Figure S1 as “% Identity to NanA” and text in the result section for further clarification (see text in quotes below).

“Here, we used an amino acid sequence of well characterized *E. coli* NanA (MG1655, GenBank: AAC76257.1) to search for homologous proteins in *F. nucleatum*. A bioinformatic analysis of 28 *F. nucleatum* proteomes spanning several subspecies identified NanA homologs in most members of the subspecies *nucleatum* and *vincentii* and some strains of *animalis*, but not in the subspecies *polymorphum* (S1 Fig).”

**Reviewer 2: 2.5** Fig. 1 Legend requires clarification with regards to the growth medium used in the experiment and as control.

**Author Response:** We thank the reviewer for this suggestion. Columbia media supplemented with Neu5Ac was used as control in this experiment. We have now added this information to the figure legend and also clarified this in the methods.

**Reviewer 2: 2.6** L.186,187. 'plateauing' sounds like of Lab jargon and may be replaced by 'reaching a plateau'

**Author Response:** We have modified the sentence in the revised manuscript as suggested by the reviewer.

**Reviewer 2: 2.7** L. 274 the term used 'somewhat different' vaginal microbiota is too vague and should be refined.

**Author Response:** Please see our response to similar comment from Reviewer 1 in 1.6.

**Reviewer 3:**

**Carl Yeoman – please note that this reviewer has waived anonymity
Reviewer 3:** Apologies for the delay, I really enjoyed reading this paper and felt it was another great and useful addition to the literature from the Gilbert lab. The manuscript describes a number of well designed and deployed experiments that examine the microbial ecology related to Fusobacterium nucleatum colonization and persistence of the vaginal microenvironment and a potential mutualism with G. vaginalis  adding important insight into the ecological interactions underlying vaginal dysbiosis and related-diseases, with particular relevance to pre-term birth. I commend the authors on the amount of work that has clearly gone into this manuscript and the well thought out set of experiments. Below are only minor thoughts and comments for consideration:

**Author:** We thank Reviewer 3 for appreciating the novelty and design of our study. We have added text and provide additional references in the manuscript for more clarity.

Minor Comments:
**Reviewer 3:** Line 54: Beware of the creep in reporting of these statistical data - available data supports a little less than 30 % of US women are affected by BV, which has since been reported as 30 % and subsequently as a third of US women. This may seem small but is actually very large.

**Author Response:** We thank the reviewer for bringing this to our notice. We have now revised this statement to reflect that ~29% of US women are affected by BV based on 2001-2004 National Health and Nutrition Examination Survey data (DOI:[10.1097/01.AOG.0000247627.84791.91](https://doi.org/10.1097/01.AOG.0000247627.84791.91)).

**Reviewer 3:** Lines 62 - 64: Aside from the grammatical error of placing the comma (which should probably be a semicolon) inside the brackets, this sentence appears to be miswritten - the healthy microbiome excluding potentially pathogenic members leads to dysbiosis?

**Author Response:** We thank the reviewer for noticing this grammatical error. We have now edited this sentence for more clarity to read as - “It is widely held that a healthy microbiome thwarts the entry of potentially pathogenic members into the community (referred to as “colonization resistance”) and that the loss of this “colonization resistance” plays a vital role in the development of vaginal dysbiosis [14].”

**Reviewer 3:** Lines 481+ In addition to oro-vaginal contact, other routes have previously been proposed, including in the context of pre-term delivery (e.g. see Cobb et al. 2017. DOI: 10.2147/IJWH.S142730) and while I don't think evidence supports a placental microbiome, of relevance to this paper is the similarities 16S studies have found between the placenta and oral microbiota (e.g. Aagaard et al. 2016. DOI: 10.1126/scitranslmed.3008599).

**Author Response:** We agree with the reviewer that hematogenous transmission of *Fusobacterium* from oral cavity to placenta has been proposed as a route for intrauterine infection. We are not discussing routes of intrauterine infection in the discussion section (our study did not directly address this). However, we agree that mention of this route is an important introductory point and have added the Cobb et al reference and a statement about the oral-hematogenous route of placental infection in the introduction. This section now reads, “*F. nucleatum is* a Gram-negative spindle-shaped obligate anaerobe that is ubiquitous in the human mouth, but also one of the most commonly isolated microorganisms from amniotic fluid of women in preterm labor [37-39]. Although the prevailing view is that *F. nucleatum* reaches the amniotic fluid through an oral-hematogenous route {Cobb et al. 2017. DOI: 10.2147/IJWH.S142730}, vaginal colonization with *F.* n*ucleatum* is also a risk factor for amniotic fluid infection and preterm birth [13].”
